# Nonthermal pulsed electric field recellularization in the duodenum for type 2 diabetes mellitus

**DOI:** 10.1016/j.vgie.2024.08.013

**Published:** 2024-08-29

**Authors:** Barham K. Abu Dayyeh, Samuel J. Asirvatham

**Affiliations:** 1 Division of Gastroenterology and Hepatology, Mayo Clinic, Rochester, Minnesota, USA; 2 Department of Cardiology, Mayo Clinic, Rochester, Minnesota, USA

## Background

The duodenum is pivotal in nutrient-sensing and iron absorption, influencing the incretin response and type 2 diabetes (T2D) pathophysiology. Dysregulated iron homeostasis and duodenal inflammation are potential therapeutic targets.^1^, ^2^, ^3^, ^4^, ^5^ Diabetes-associated duodenal changes are implicated in the pathogenesis of type 2 diabetes and represent a therapeutic target.^6^, ^7^, ^8^ Thermal modalities for duodenal mucosal ablation have several limitations, including the inability to regenerate deeper submucosal duodenal targets, heat-mediated degradation of the extracellular matrix, and the inherent risks associated with thermal injury.^9^^,^^10^ Endoscopic recellularization via electroporation therapy (ReCET) is a novel, nonthermal approach to induce intestinal cell regeneration using pulsed electric fields.

## The ReCET device and procedure

**Figure 1:**
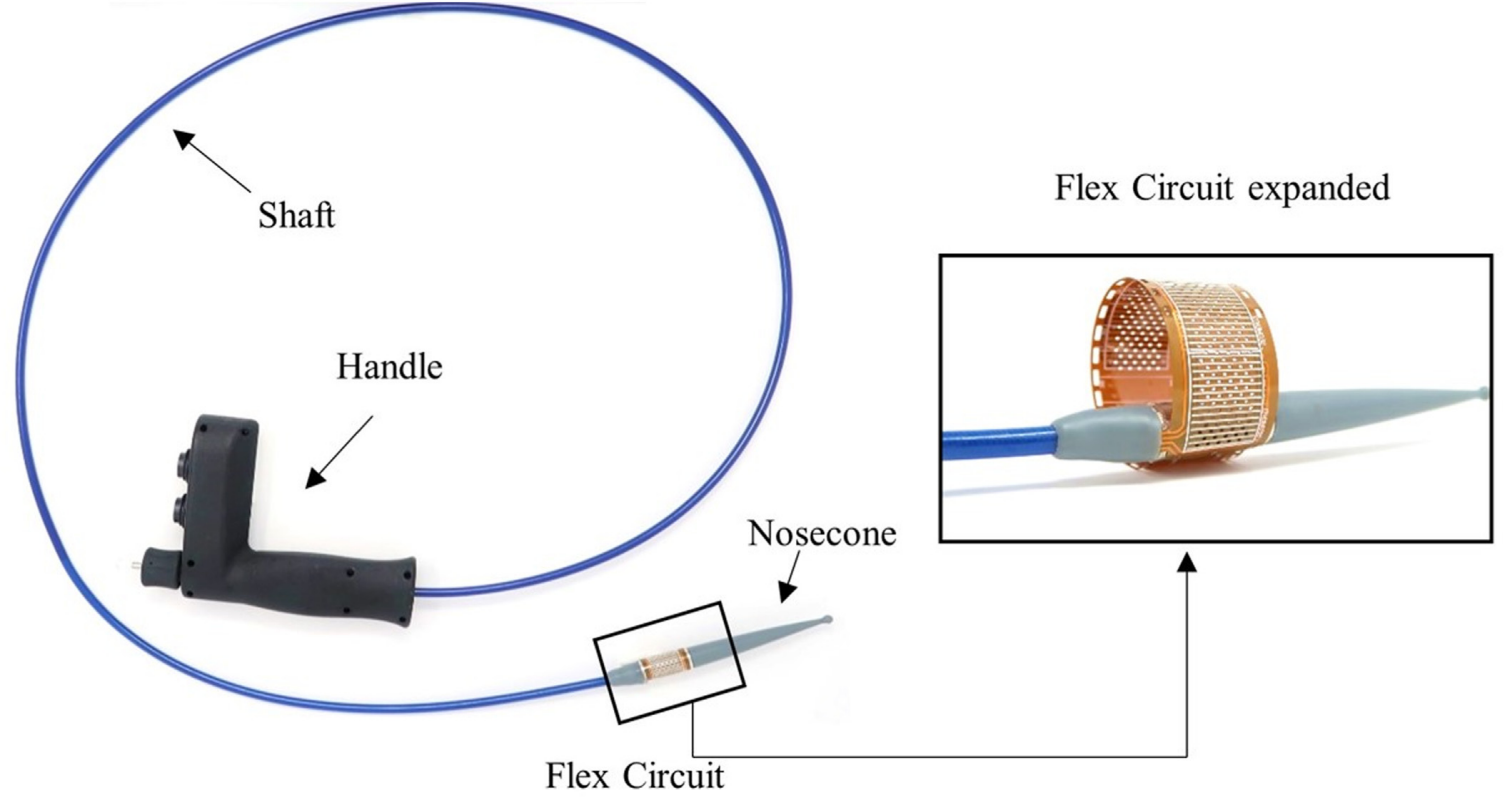
An illustration of the catheter and flexible circuit used for the application of pulsed electric field in the duodenum.

**Figure 2:**
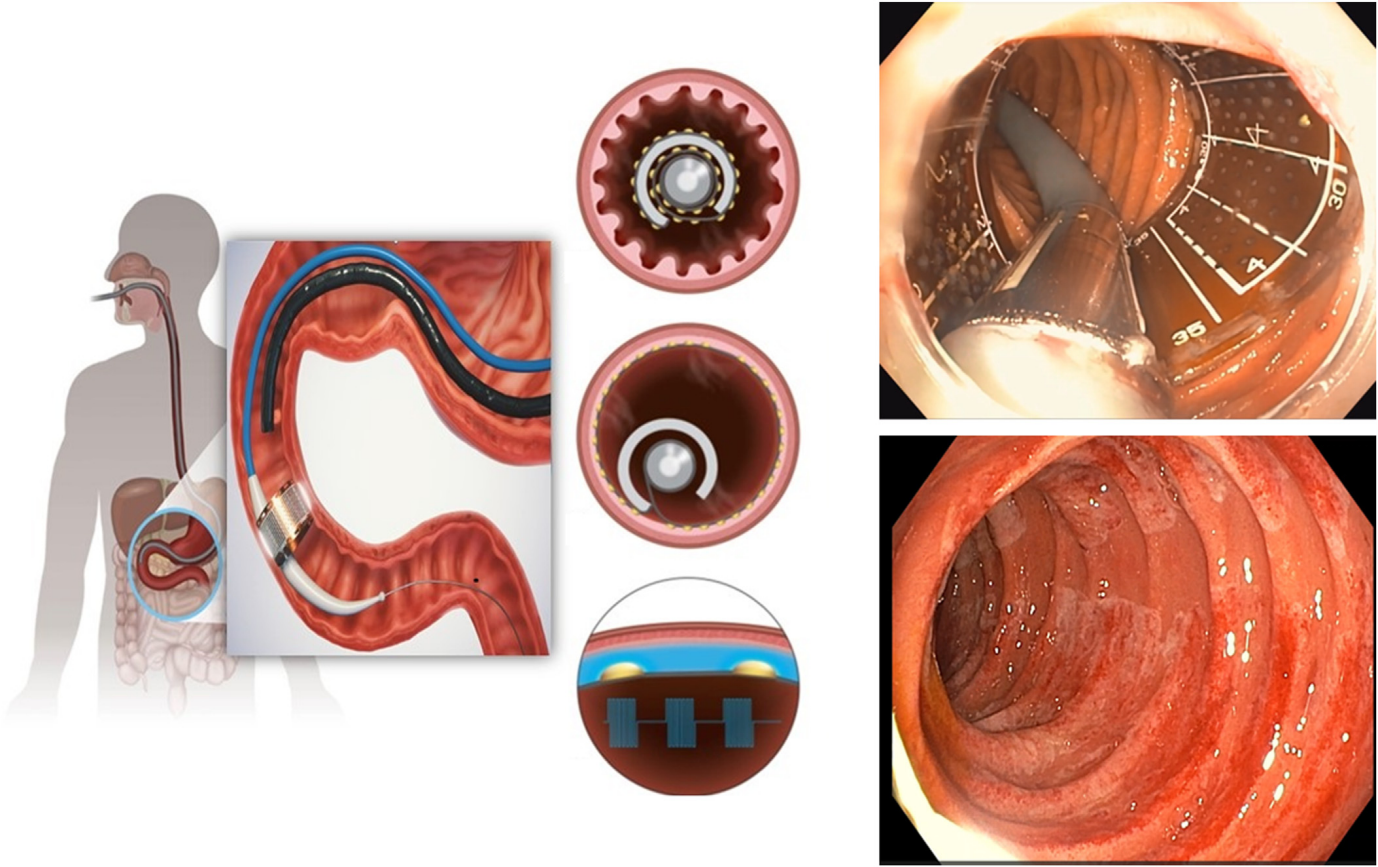
Application of pulsed electric field in the duodenum. The flexible circuit is positioned in the duodenum before expansion. The flexible circuit is expanded, engaging the duodenal wall and optimizing it for the delivery of the pulsed electric field. This showcases the micropulsed electrical field, offering precise depth control through the technology, and demonstrating a circumferential, homogeneous, nonthermal treatment effect on the duodenal wall.

The ReCET system by Endogenex (Plymouth, Minn, USA), integrates a pulsed electric field generator, a disposable catheter, and connection cables (Fig. 1). It uses proprietary algorithms for precise energy dose and waveform control, targeting specific treatment depths. The catheter features a variable-stiffness shaft, a center lumen for up to 0.038-inch guidewires, a therapeutic segment with electrode arrays for energy delivery, an atraumatic tip, and a handle. The flexible circuit board in the therapeutic segment is 2 cm long, adjustable up to 45 mm to suit various duodenal sizes and optimize contact for effective energy application. Procedures involve fluoroscopic and endoscopic guidance, placing the flexible circuit 2 cm beyond the ampulla of Vater, and expanding it for duodenal wall contact. Pulsed electric field therapy is then administered (Fig. 2). Post-therapy, the circuit is retracted and repositioned for further treatment. A case study of its application in a 68-year-old man with a body mass index of 34 kg/m^2^, long-standing T2D, and suboptimal glycemic control (hemoglobin A1c 8.4%) despite multiple medications is presented (Video 1, available online at www.videogie.org).

## Conclusions

The ReCET procedure represents a significant advancement in T2D treatment, focusing on the duodenum’s role in nutrient-sensing and inflammation as an intestinal cellular regeneration therapy. It has demonstrated promising results in a patient with long-standing, poorly controlled diabetes. The potential of ReCET as a disease-modifying therapeutic tool underscores the necessity for further research of its clinical application in the management of diabetes.

## Disclosure

Dr Abu Dayyeh is a consultant to Boston Scientific, Medtronic, and Olympus and co-inventor of the ReCET technology licensed by the Mayo Clinic to Endogenex. Dr Asirvatham is co-inventor of the ReCET technology licensed by the Mayo Clinic to Endogenex.

## Supplementary data

Video 1 This video provides relevant pathophysiology on the role of duodenal-signaling dysregulation in type 2 diabetes and demonstrates the application of pulsed electric field in the duodenum as a novel regenerative therapy for type 2 diabetes.
